# Active Compounds of *Panax ginseng* in the Improvement of Alzheimer’s Disease and Application of Spatial Metabolomics

**DOI:** 10.3390/ph17010038

**Published:** 2023-12-26

**Authors:** Meng Zhang, Huazhou Niu, Qingqing Li, Lili Jiao, Hui Li, Wei Wu

**Affiliations:** Jilin Ginseng Academy, Changchun University of Chinese Medicine, Changchun 130117, China; zhangmenghh945@163.com (M.Z.); niuhuazhou95@163.com (H.N.); liqqsunny@126.com (Q.L.); jiaoaj@hotmail.com (L.J.)

**Keywords:** *P. ginseng*, chemical component, ginsenoside, Alzheimer’s disease, signaling pathways, spatial metabolomics

## Abstract

*Panax ginseng* C.A. Meyer (*P. ginseng*) is one of the more common traditional Chinese medicines (TCMs). It contains numerous chemical components and exhibits a range of pharmacological effects. An enormous burden is placed on people’s health and life by Alzheimer’s disease (AD), a neurodegenerative condition. Recent research has shown that *P. ginseng*’s chemical constituents, particularly ginsenosides, have a significant beneficial impact on the prevention and management of neurological disorders. To understand the current status of research on *P. ginseng* to improve AD, this paper discusses the composition of *P. ginseng*, its mechanism of action, and its clinical application. The pathogenesis of AD includes amyloid beta protein (Aβ) generation and aggregation, tau protein hyperphosphorylation, oxidant stress, neuroinflammation, mitochondrial damage, and neurotransmitter and gut microbiota disorders. This review presents the key molecular mechanisms and signaling pathways of the active ingredients in *P. ginseng* involved in improving AD from the perspective of AD pathogenesis. A *P. ginseng*-related signaling pathway network was constructed to provide effective targets for the treatment of AD. In addition, the application of spatial metabolomics techniques in studying *P. ginseng* and AD is discussed. In summary, this paper discusses research perspectives for the study of *P. ginseng* in the treatment of AD, including a systematic and in-depth review of the mechanisms of action of the active substances in *P. ginseng*, and evaluates the feasibility of applying spatial metabolomics in the study of AD pathogenesis and pharmacological treatment.

## 1. Introduction

*Panax ginseng* C.A. Meyer (*P. ginseng*) is a perennial herb belonging to the family Araliaceae and the genus *Panax. P. ginseng* has been used for thousands of years; not only does it possess pharmacological properties, but it also has few side effects. It has been used worldwide as a functional food and complementary medicine, among other purposes [1]. *P. ginseng* comprises many biologically active components, including ginsenosides, gintonin, polysaccharides, peptides, glycoconjugates, and other compounds. The most commonly studied bioactive components claimed to have health benefits are ginsenosides [2]. Ginsenosides have been extensively used for many years to treat various diseases, especially neurodegenerative diseases, including AD. In addition, other active ingredients of *P. ginseng*, including gintonin and ginseng polysaccharides, have been found to have certain therapeutic effects on nervous system diseases.

AD is a neurodegenerative disease that manifests as a decline in cognitive and memory function. Several factors, including age, sex, and environmental factors, play a role in the development of the disease. It is mostly seen in people over 65 years old. In recent years, AD has attracted extensive research attention as a multifactorial neurodegenerative disease [3]. The pathogenesis and etiology of AD, a relatively common neurodegenerative disease, have not been fully elucidated, and there is a lack of effective treatments [4]. In addition, AD is thought to begin decades before symptoms become apparent [5]. Thus, for the sake of early diagnosis, prevention, and therapy, it is crucial to understand the pathogenesis of AD.

The amyloid cascade and tau proteins are the principal subjects of research with regard to the processes of AD [6]. Senile plaques (SPs), formed by the aggregation of amyloid beta protein (Aβ) and neurofibrillary tangles (NFTs), formed by the hyperphosphorylation of tau proteins, are two hallmark pathologies of AD. In addition, it has been found that the development of AD is accompanied by additional pathological mechanisms. Therefore, multitarget and multichannel therapies should be highlighted as emerging strategies for AD treatment. Moreover, there is growing evidence that components of *P. ginseng* are important in improving AD. The active ingredients in *P. ginseng* can also improve AD by acting on different signaling pathways and pathogenic mechanisms. In addition, *P. ginseng* extracts can be used in combination with other drugs for better therapeutic efficacy [1].

Metabolomics is a systematic approach for comprehensively analyzing small molecules in biological samples, and its application can provide clinically favorable biomarkers [7]. The application of metabolomics is important for the diagnosis of diseases, the study of disease mechanisms, and the selection of drugs [8]. Mass spectrometry imaging (MSI) techniques combine mass spectrometry with microscopic imaging technology to not only provide large amounts of untargeted chemical information, but also identify the spatial distribution of a large number of molecules [9,10]. Common mass spectrometry imaging technologies include matrix-assisted laser desorption ionization (MALDI), secondary ion mass spectrometry (SIMS), desorption electrospray (DESI), and air-flow-assisted desorption electrospray ionization (AFADESI). Spatial metabolomics with the use of mass spectrometry imaging can identify biological pathways of disease based on abnormal distribution and abundance of metabolites [11]. All of this suggests that spatial metabolomics is expected to provide favorable technical support for studying the complex pathophysiology of AD.

Although studies on the effects of *P. ginseng* on AD have been summarized many times [1,12], to more systematically elucidate the mechanisms of these effects, in this review, we constructed several comprehensive signaling pathway networks for each mechanism of action. In addition to providing an overview of commonly described mechanisms, this paper also emphasizes the role of gut microbes. Finally, this review summarizes recent studies and suggests the feasibility of applying spatial metabolomics to the study of *P. ginseng* for the treatment of AD.

## 2. Active Ingredients of *P. ginseng*

**Figure 1:**
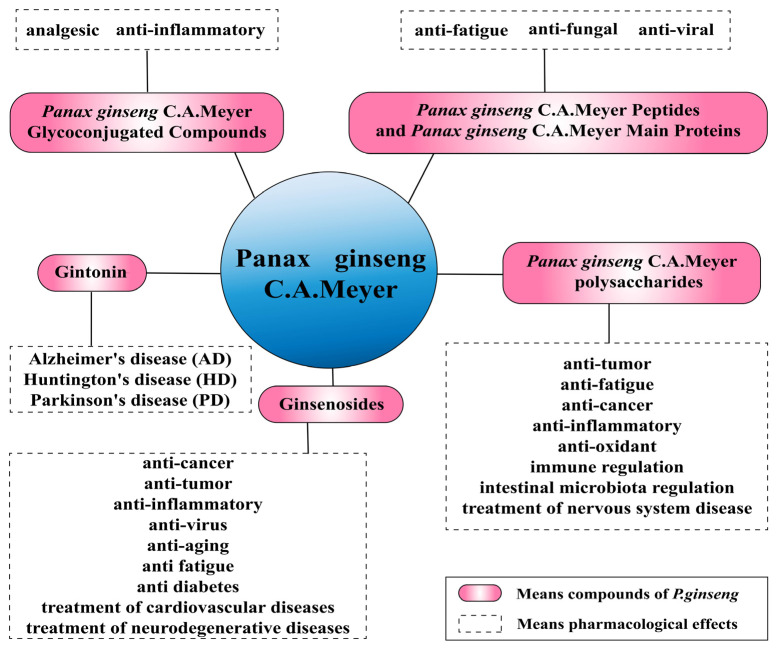
Active ingredients and pharmacological effects of *P. ginseng*.

*P. ginseng* contains many bioactive and potentially effective therapeutic compounds. Wang et al. used the TCMSP database to screen the active ingredients of *P. ginseng* and found that a total of 190 chemical components have been identified, among which 22 are active components [13]. Numerous studies have found that the main types of compounds in *P. ginseng* include ginsenosides, gintonin, polysaccharides, peptides, and glycoconjugates. These active ingredients have been found to have various pharmacological effects and can provide good therapeutic benefits for different diseases (Figure 1). The various components and their active roles were reviewed in order to provide a theoretical basis for better development and utilization of *P. ginseng*.

### 2.1. Ginsenosides

**Figure 2:**
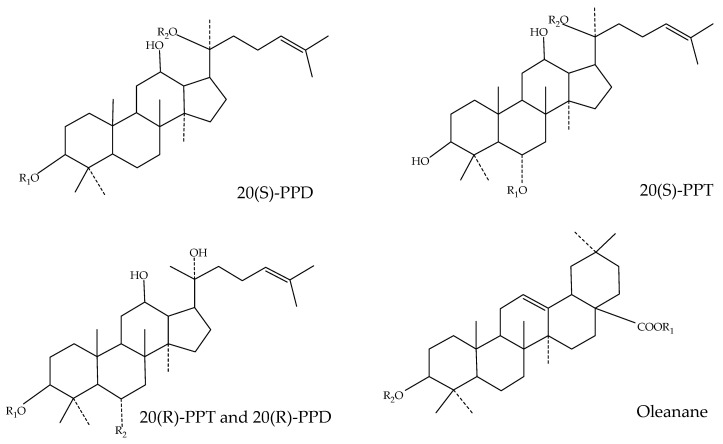
The chemical structures of ginsenosides (R1 and R2 refer to hydrogen or different sugar group substitution; different ginsenosides have different R1 and R2 in their structural formula).

Ginsenosides are the main active ingredient in several parts of *P. ginseng*. Ginsenosides are steroidal glycosides. The reported ginsenosides are mainly classified based on the type of saponin, as well as the number, type, and site of glycosyl units. They can be classified as either dammarane or oleanolic acid based on the glycosidic backbone: a tetracyclic backbone with a sugar linkage portion or a pentacyclic backbone with oleanolic acid (OA) and C17 side chain variant (C17SCV) isoforms, respectively. According to the position of the sugar connection, the dammarane skeleton is further classified as a protopanaxadiol (PPD) or protopanaxatriol (PPT) ginsenoside, whose sugar components are connected at the third or sixth position of the triterpenoid structure, respectively [14]. The structural formulae of ginsenosides are shown in Figure 2 [15]. A total of 170 ginsenosides have been identified from different parts of *P. ginseng*, including 59 OA, 42 PPD, 65 PPT, and 17 C17CSV types. The composition of ginsenosides in different parts is significantly different. A total of 16 types of ginsenosides are commonly present in all tissues, including 9 PPD, 6 PPT, and 1 OA types (and no C17SCV). Among them, ginsenosides Rb2, Rb1, Rc, Rd, Re, Rf, Rg1, and Ro comprise the highest content in *P. ginseng*, accounting for more than 70% of total saponins. In addition, malonyl saponins containing malonyl groups have also been detected in different parts of *P. ginseng*. In the roots, rhizomes, leaves, stems, and fruits, protopanaxadiol-type ginsenosides predominate, whereas higher proportions of malonyl- and C17SCV-type ginsenosides are found in the flowers and buds [2].

Each type of ginsenoside has different pharmacological effects. As the main active ingredients in *P. ginseng*, ginsenosides exhibit significant pharmacological activities, such as anticancer [16], antitumor [17], anti-inflammatory [18], antivirus [19], antiaging [20], antifatigue, and anti-diabetes [21] activities, and can be used in the treatment of cardiovascular diseases [22,23] and neurodegenerative diseases [24,25], including AD [26].

Therefore, increasing the production of ginsenosides and developing more types of ginsenosides are important in the treatment of various diseases. Traditional extraction methods are time-consuming and have low yields; alternative biotechnologies, such as hair root culture, polyploidy, cell suspension culture, protoplast culture, conventional tissue culture, and in-vitro mutagenesis, have been proposed to address these issues and greatly increase the yield of ginsenosides [27]. In addition, the method of using microbial hosts has also been proposed for more efficient and selective production of ginsenosides [28]. Rare ginsenosides also have various biological activities, but their content is extremely low in *P. ginseng*. Different extraction methods can have an impact on the structure of ginsenosides [29]. An effective combination biotechnology method, which includes tissue culture, fixation, and hydrolysis, has been developed for the production of rare ginsenosides, including Rh2 [30]. In the process of ginsenoside biosynthesis, glycosyltransferases play an important role in forming diverse structures and have biological activity by transferring various sugar parts into ginsenosides. In addition, UDP glycosyltransferases (UGTs) were discovered to help produce rarer ginsenosides [31].

### 2.2. Gintonin

Gintonin, a glycoprotein complex isolated from *P. ginseng*, contains three lipid-derived G-protein-coupled receptor ligands: lysophosphatidylinositol (LPI), lysophosphatidic acid (LPA), and linoleic acid (LA). They are the main functional components of gintonin.

Gintonin plays an important role in the treatment of neurodegenerative diseases, including AD. Gintonin has effects against disorders such as AD, Parkinson’s disease (PD), and Huntington’s disease (HD). It can regulate the levels of neurotransmitters, such as acetylcholine, dopamine, norepinephrine, and serotonin, exert antioxidant and anti-inflammatory effects, and induce autophagy to reduce Aβ production. Gintonin plays an ameliorative role in degenerative diseases through the above mechanisms [32]. In addition, gintonin induces rapid and transient opening of the BBB, enhancing the delivery of molecules to the brain. This effect could be used to enhance the therapeutic effect of drugs on brain diseases [33]. Gintonin can facilitate the delivery of AD treatment drugs to the brain, according to one study [34]. This means that gintonin is essential for AD treatment.

### 2.3. Ginseng Polysaccharides

Ginseng polysaccharides (GPs) are a class of bioactive compounds present in *P. ginseng*, which has so far yielded roughly 80 different kinds of polysaccharides, both acidic and neutral. The polysaccharides in *P. ginseng* are mostly neutral, primarily amyloid mixes, dextrans, and arabinogalactans. A small percentage are acidic; these are pectin-containing rhamnose and homo galacturonic acid [35].

Studies have shown that ginseng polysaccharides have antitumor [36], antifatigue [37], anticancer [38], anti-inflammatory [39], antioxidant [40], immune regulation [41], and intestinal microbiota regulation activities [42], and can be used in the treatment of nervous system diseases [43].

### 2.4. Ginseng Peptides and Main Proteins

The primary proteins found in *P. ginseng* are known as ginseng main proteins (GMPs). The study of low-molecular-weight parts of proteins, such as endogenous peptides, protein fragments, and protein degradation products, is called peptidomics [44]. Peptides have a simpler structure than proteins, making them easier to examine, and they can mimic the creation, processing, and destruction of proteins.

*P. ginseng* proteins contain antiviral, antifungal, and antifatigue properties. The characteristics and roles of precursor proteins can be better understood using peptide fragments. Peptides are anticipated to be potential molecules for evaluating the potency of medicinal plants [45].

### 2.5. Ginseng Glycoconjugates

Glycoconjugates are compounds that covalently connect one or more monosaccharide or oligosaccharide units with non-carbohydrate parts (proteins, lipids, etc.).

As biological molecules, glycoconjugates participate in regulating various life activities in organisms, and they have anti-inflammatory and analgesic properties. Luo et al. used *P. ginseng* as the raw material, used 85% ethanol reflux for extraction, and then purified the extract with a G-15 column of silk fatty amide to prepare ginseng glycopeptides (Gg). They found that the ginseng glycopeptides had anti-inflammatory and analgesic effects [46].

## 3. Pharmacological Effects of Active *P. ginseng* Compounds on Improving AD

AD is caused by many factors. The occurrence and development of AD result from the joint interaction of various pathogenic components and multiple targets. Many studies have shown that various active substances in *P. ginseng*, especially ginsenosides, can alleviate AD by regulating signaling pathways related to mechanisms of the disease. Previous studies intensively investigated the mechanism of *P. ginseng* in improving Aβ aggregation and tau hyperphosphorylation involving more signaling pathways. Research on neuroinflammation, oxidative stress, and neurotransmitters is also increasing. It is worth noting that the contribution of mitochondrial damage and intestinal microbe disorders to AD has also received extensive attention in recent years. This review summarizes the multiple pathways involved in these pathogenic mechanisms, with the expectation of providing theoretical support for the use of *P. ginseng* in AD treatment.

### 3.1. Interference of Aβ Generation and Aggregation

**Figure 3:**
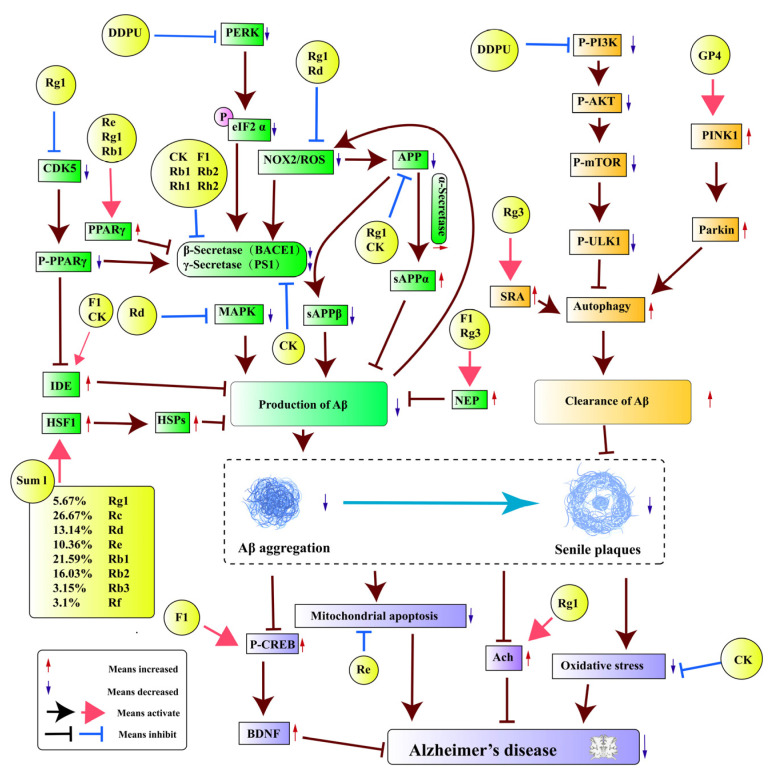
Signaling pathways related to Aβ accumulation.

Aβ amyloid plaques (senile plaques, SPs) produced by aggregation are important markers of AD [47,48]. Several peptides with 36–43 amino acid residues go under the generic name Aβ. Two peptides, with 40 and 42 amino acid residues, represent the two major species (Aβ1-40 and Aβ1-42) [49]. Aβ aggregation is neurotoxic, leading to membrane rupture, abnormal cell signaling, and organelle dysfunction [50]. In addition, it induces a series of deleterious responses, such as the induction of tau hyperphosphorylation, neuroinflammation, mitochondrial apoptosis, and synaptic dysfunction, which can further contribute to AD. Among the pathogenic mechanisms of *P. ginseng* in AD, the Aβ protein, which contains the largest number of signaling pathways, is the most widely studied. These signaling pathways mainly play a role in reducing Aβ production and increasing Aβ clearance, as shown in Table 1 and Figure 3.

#### 3.1.1. Interference of Aβ Production by Regulating APP through Multiple Signaling Pathways

Amyloid precursor protein (APP) can be cleaved into Aβ by β-secretory and γ-secretase enzymes, or cleaved into secretory APP α (sAPPα) by α-secretase to block Aβ production. Thus, abnormal processing of APP is a key factor in Aβ accumulation. Research has shown that the active substances in *P. ginseng* can increase the expression of α-secretory enzymes or reduce the expression of β-secretory and γ-secretory enzymes through different signaling pathways, or reduce the expression of APP to further reduce the amount of Aβ, contributing to improving AD.

Presenilin 1 (PS1) is the key catalytic component of γ-secretase, and Beta-site APP-cleaving enzyme 1 (BACE1) is the key component of β-secretase. Therefore, inhibition of PS1 and BACE1 expression is a key target to block Aβ generation. A variety of active compounds in *P. ginseng* have this effect. Karpagam et al. [54] found that the ginsenosides CK, F1, Rh1, and Rh2 all have the potential to inhibit BACE1 expression. Choi et al. also found that Rb1 and Rb2 potentially have inhibitory activity against BACE1 [60]. The ginsenoside CK was found to not only reduce the expression of APP, but also inhibit the expression of BACE1 and PS1 [51]. The compound 1-(3,4-dimethoxyphenethyl)-3-(3dehydroxyl-20(s)-protopanaxadiol-3b-yl)-urea (DDPU) is one of the derivatives of PPD. DDPU can be used to decrease BACE1 protein levels by inhibiting the activation of protein kinase RNA-like endoplasmic reticulum kinase (PERK) and decreasing the phosphorylation of eukaryotic translation initiation factor-2α (eIF2 α) [61]. Similarly, the ginsenoside Rg1 was found to reduce Aβ production by inhibiting APP production and modulating α-, β-, and γ-secretase activity [65]. Quan et al. reported that the ginsenoside Rg1 can significantly reduce the expression of cyclin-dependent kinase 5 (CDK5), inhibit the phosphorylation of peroxisome proliferator-activated receptor y (PPARγ), and downregulate the expression of BACE1, decreasing Aβ levels [52]. In contrast, another study found that Rg1 decreased Aβ levels by increasing PPARγ expression in the hippocampus [65]. In addition, the ginsenosides Re [59] and Rb1 [53] have also been found to reduce Aβ production by activating PPARγ. Interestingly, Aβ deposition was shown to increase NADPH oxidase 2 (NOX2) expression and reactive oxygen species (ROS) generation, which further upregulated the expression of APP and BACE in APP/PS1 mice, resulting in a vicious circle. However, Rg1 treatment significantly decreased NOX2 expression and ROS production, thus breaking this vicious circle [57].

#### 3.1.2. Direct Interference of Aβ Production through Various Signaling Pathways

Aβ aggregation may also be directly reduced through different signaling pathways by the active compounds in *P. ginseng*.

Insulin-degrading enzyme (IDE) and Neprilysin (NEP) are important Aβ-degrading enzymes that can reduce the production of Aβ. Various active compounds in *P. ginseng* can reduce Aβ production by increasing the activity of IDE and NEP. The ginsenosides CK [51] and Rg1 [65] can increase IDE activity and reduce Aβ expression. Rg1 can increase the expression of IDE, by reducing the expression of CDK5, inhibiting the phosphorylation of PPARγ [52]. In addition, as a metabolite of Rg1, the ginsenoside F1 enhances the expression of IDE, as well as NEP, in vitro and in vivo [55]. Meanwhile, the ginsenoside Rg3 can also enhance NEP expression [56]. Additionally, by inhibiting the Mitogen-Activated Protein Kinase (MAPK) signaling pathway and decreasing ROS levels, the ginsenoside Rd is able to lower the amounts of Aβ [58]. It was found that the ginsenoside complex Sum I can increase the expression of heat shock proteins (HSPs) by upregulating the expression of heat shock factor 1 (HSF-1). This prevents the generation of misfolded or aggregated Aβ and accelerates its breakdown [26].

#### 3.1.3. Interference of Aβ Accumulation by Increasing Aβ Clearance

One of the key processes for the clearance of intracellular and extracellular Aβ is macroautophagy/autophagy [66]. Reducing Aβ aggregation by increasing autophagy is also an important way that *P. ginseng*’s active substances resist AD.

It has been reported that DDPU suppresses the phosphorylation of Phosphoinositide 3-kinase (PI3K), v-akt murine thymoma viral oncogene homologue (AKT), mammalian target of rapamycin (mTOR), and UNC-51-like kinase 1 (ULK1) to reduce Aβ [61]. The ginsenoside Rg3 could increase the expression of scavenger receptor type A (SRA) and upregulate the uptake of Aβ42 in an in-vitro model, as recently reported by Ahn et al. [62]. Furthermore, a 4.7 kDa ginseng-derived polysaccharide (GP4) was found to induce autophagy and reduce Aβ aggregation, possibly through activation of PTEN-induced kinase 1 (PINK1)/Parkin RBR E3 ubiquitin-protein ligase (Parkin) [63].

#### 3.1.4. Improving AD by Modulating Aβ Downstream Mechanisms

Aβ aggregation further regulates downstream signaling pathways and promotes the generation of other pathogenic mechanisms. Active substances in *P. ginseng* have been found to not only regulate the downstream pathways of Aβ, but also improve other pathological conditions caused by Aβ as a way to combat AD.

Brain-derived neurotrophic factor (BDNF) and cyclic adenosine monophosphate (cAMP) response element binding protein (CREB) are the key downstream mediators of Aβ toxicity. It has been suggested that Aβ, by decreasing CREB phosphorylation, decreases BDNF expression and, consequently, has a toxic effect in AD [20]. The ginsenoside F1 plays a therapeutic role in AD by enhancing the expression of a phosphorylated form of CREB in the hippocampus and increasing the expression of BDNF [64]. In addition, the ginsenoside Re can improve Aβ-aggregation-induced mitochondrial damage [67]. The ginsenoside Rg1 was found to increase the content of acetylcholine in AD model rats induced by Aβ aggregation [68]. The ginsenoside CK can prevent oxidative stress caused by Aβ aggregation, playing a neuroprotective role [51].

### 3.2. Inhibition of Tau Hyperphosphorylation

One of the established pathogenic components of Alzheimer’s disease is the hyperphosphorylation of tau proteins, which is linked to microtubules. The active compounds in *P. ginseng* inhibit the hyperphosphorylation of tau proteins through various signaling pathways, thus preventing the generation of NFTs and the destruction of microtubules.

#### 3.2.1. Manifestations of Tau Hyperphosphorylation

Neuronal structure, functionality, and plasticity are significantly influenced by the microtubule system. Changes in the tissue and structure of microtubules and microtubule regulatory proteins (such as the microtubule-associated tau protein) are characteristic of a variety of neurodegenerative diseases [69]. The deposition of tau aggregates is a pathological hallmark of AD that is closely linked both spatially and temporally to the emergence of neurodegeneration and clinical symptoms [70]. Normally, tau controls the assembly and transport of microtubules (MTs). When tau is hyperphosphorylated in AD, it separates from the MT assembly, causing destabilized MTs and impaired axonal transport. Paired helical filaments (PHFs), which are filamentous structures made of phospho–tau aggregates, combine to create aggregates of insoluble NFTs. In addition, there is growing evidence that tau hyperphosphorylation may be caused by Aβ, which eventually causes MT integrity to be compromised [71].

#### 3.2.2. Inhibition of Tau Hyperphosphorylation by Active Compounds of *P. ginseng*

**Figure 4:**
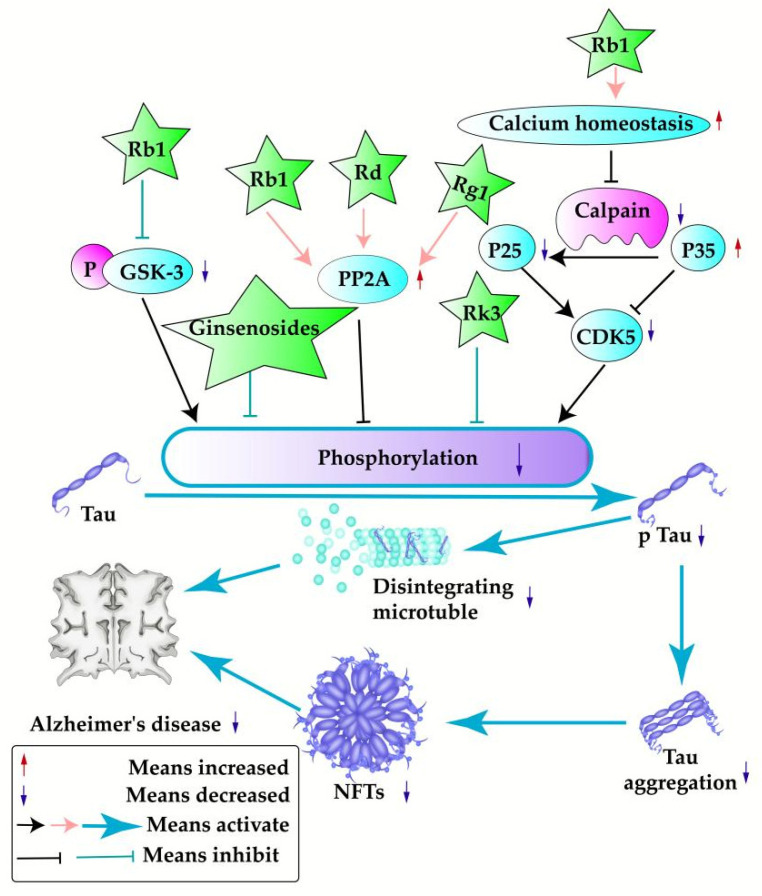
Signaling pathways related to tau hyperphosphorylation.

The active substances of *P. ginseng* regulate tau-related signaling pathways, as shown in Table 2 and Figure 4.

Glycogen synthase kinase (GSK-3) and protein phosphatase 2A (PP2A) are the main enzymes regulating tau phosphorylation. GSK-3 has two isoforms: alpha and beta. The ginsenoside Rb1 can exert neuroprotective effects by reducing pGSK-3 levels and increasing PP2A levels, preventing tau hyperphosphorylation [72]. In addition, it was proven that the ginsenosides Rd [73] and Rg1 [74] exert neuroprotective effects by preventing tau hyperphosphorylation by increasing the level of PP2A. CDK5 is one of the main protein kinases of tau, and the conversion of p35 to p25 can lead to the dislocation of CDK5. The ginsenoside Rb1 can effectively maintain intracellular calcium homeostasis and block the activation of calpain, prevent calpain-mediated transformation of P35 to P25, and reduce the activity of CDK5 [75]. Furthermore, Rk3 and ginsenosides have the effect of inhibiting tau protein hyperphosphorylation, but the relevant therapeutic mechanisms have not been elucidated [57,76,77].

### 3.3. Regulation of Neurotransmitter Levels

It has been found that another important mechanism of AD is changes in neurotransmitter levels. Neurotransmitters carry information across neuronal synapses and neuromuscular junctions [78]. AD is often accompanied by changes in the levels of neurotransmitters, including cholinergic, glutamatergic, GABAergic, serotonin, noradrenergic, and histaminergic changes. Changes in neurotransmitter levels have different effects on cognitive and memory function through different methods of action, leading to nervous system diseases. Therefore, regulating neurotransmitter levels and ensuring their stability is an effective treatment direction. In the past, studies on *P. ginseng*’s modulation of neurotransmitters in AD were mainly focused on ACh, with relatively few studies on other neurotransmitters, as shown in Table 3.

#### 3.3.1. Regulation of ACh Levels

Acetylcholine (ACh), an important neurotransmitter in the cholinergic system, is one of the most widely studied neurotransmitters as a countermeasure against AD. A decreased acetylcholine level is an important factor in the occurrence of AD. It was found that Rh2, a rare ginsenoside in *P. ginseng*, can have neuroprotective effects by regulating cholinergic transmission and inhibiting oxidative stress, which had a therapeutic effect on a mouse model with scopolamine-induced AD [79]. In addition, it was found that the ginsenoside Rg1 could increase the amount of ACh in the hip cells of AD mice [68]. The ginsenoside Rb1 can promote the release of acetylcholine in the central nervous system [82]. The ginsenoside Re can increase the levels of extracellular acetylcholine [83]. ACh can be degraded by two kinds of cholinesterase (CHS): acetylcholinesterase (AChE) and butyrylcholinesterase (BChE). Therefore, the regulation of AChE and BChE plays a certain role in the treatment of AD, as reported by Kamecki et al. [87]. It has been found that the ginsenosides Rb1, Rb2, Rc, Re, Rg1, and Rg3 have an inhibitory effect against AChE and BChE. Among them, Re has the most significant AChE-inhibitory activity and Rg3 has the most significant BChE-inhibitory activity [60,84]. In addition, Ginseng stem and leaf saponins (GSLSs) can improve the cognitive impairment of patients with AD. Through activity screening and analysis of GSLSs, 31 ginsenosides were found, of which 27 compounds have acetylcholine–enzyme binding activity, and 11 were identified via the enzyme method. This result suggests that the inhibition of AChE may be a potential mechanism by which GSLSs improve AD [80]. It was found experimentally that PPT can improve the cognitive and memory impairment caused by scopolamine and plays a certain role in the treatment of AD. The mechanism may be that PPT inhibits the activity of acetylcholinesterase, increasing the level of acetylcholine [81]. In addition, ChAT is a key enzyme in the synthesis of ACh. Thus, the regulation of ChAT levels is also an important mechanism for improving AD. Gintonin has been found to play an ameliorative role in AD by upregulating choline acetyltransferase (ChAT) expression, increasing ACh levels, and decreasing AChE expression [85].

#### 3.3.2. Regulation of Other Neurotransmitter Levels

The active substances in *P. ginseng* can also regulate the levels of other neurotransmitters, contributing to improving AD. Amino acids, especially glutamic acid (Glu) and aspartic acid (Asp), are the main excitatory neurotransmitters in the hippocampus and cortex of the brain. Excitotoxicity caused by excitatory amino acid neurotransmitters can lead to various neurodegenerative diseases. GABA is an important inhibitory neurotransmitter in the central nervous system. Reduced GABA levels may be a potential cause of behavioral and psychological symptoms in AD. Glutamatergic and GABAergic neurotransmitters involved in Aβ-induced damage have the opposite effect. Extra cellular vesicles (EVs) are the main transmitters of intercellular information. It was found that the small EVs (sEVs) of neurons are regulated by a balance in neurotransmitter levels, and sEVs released by GABA-treated neurons reduce Aβ-induced injury, while sEVs released by neurons treated with glutamate aggravate Aβ-induced toxicity [88]. Changes in glutamatergic, GABAergic, and cholinergic levels affect the dysfunction of neural activity and the distribution of amyloid and tau, and then induce AD. Among them, the GABAergic level has the greatest impact on cognitive ability, as recently reported by Khan et al. [89]. It was found that ginsenosides can increase the levels of GABA, acetylcholine, and dopamine and reduce the levels of glutamate and aspartate in the hippocampus and cortex, and increase the levels of glycine and serotonin (5-HT) in the blood. Therefore, it can be assumed that ginsenosides play a role in improving AD by modulating a variety of neurotransmitters [76]. In addition, the ginsenoside Re can improve AD by increasing levels of extracellular dopamine (DA) [83]. Mitochondrial dysfunction can lead to abnormal amino acid metabolism. Abnormal metabolism of amino acids, including alanine (Ala), aspartate (Asp), glutamate (Glu), D-glutamine (D-Gln), D-glutamate (D-Glu), and tryptophan (Trp), has been repeatedly reported in the pathogenesis of AD. Rg3 may regulate the disordered amino acid metabolism of AD [86].

### 3.4. Anti-Oxidative Stress and Anti-Inflammation

It has been reported that the occurrence of neurodegenerative disease is often accompanied by oxidative stress [90] and neuroinflammation [91]. Various active compounds in *P. ginseng* have been found to improve AD by inhibiting oxidative stress and neuroinflammation, as shown in Table 4.

#### 3.4.1. Anti-Oxidative Stress

**Figure 5:**
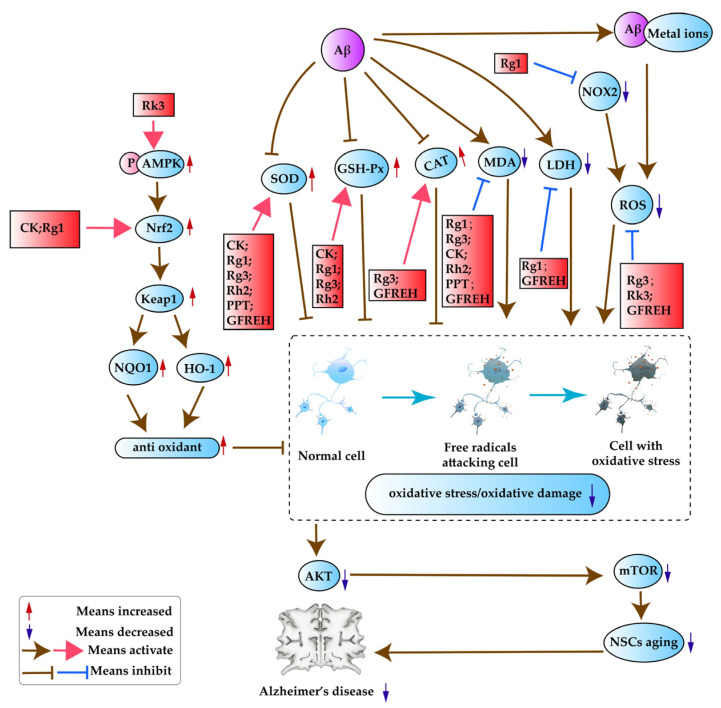
Signaling pathways related to oxidative stress.

Oxidative stress is an important pathogenic factor in the development of AD. In addition to the effects on multiple signaling pathways, the effects of *P. ginseng* on oxidative stress include the scavenging of ROS and the modulation of multiple oxidoreductases, as shown in Figure 5.

Excess ROS generation is the main cause of oxidative stress. The primary ROS source in the brain is NOX2. Aβ in combination with some metal ions, such as Cu(I/II) and Fe(II/III), causes damage to the steady state of redox and produces ROS, causing damage to neurons [50]. The ginsenoside Rg1 can reduce the production of ROS, mediated by the inhibited expression of NOX2 [57]. By scavenging ROS, it was reported that Rg3 may have protected rats from the oxidative stress of AD [86]. It was found that Rk3 can attenuate intracellular ROS production [77]. Ginseng fibrous root enzymatic hydrolysate (GFREH) was first prepared by digesting fibrous ginseng roots with alkaline protease. GFREH can reduce ROS levels in worms and has antioxidant activity [94]. The ginsenoside Rg1 can downregulate the Akt/mTOR signaling pathway by reducing the production of ROS to suppress NSC aging, thus exerting a neuroprotective effect [93]. Heme oxygenase 1 (HO-1) is the main antioxidant protein in the body; it promotes the degradation of heme and the production of biliverdin, and eliminates free radicals accumulated by an oxidation–reduction imbalance, thereby exerting an antioxidant effect. It has been found that Rk3 has antioxidant activity by activating nuclear factor erythroid 2-related factor 2 (Nrf2) and its downstream proteins, including HO-1 and NAD(P)H: quinone oxidoreductase 1 (NQO1), through increased phosphorylation of adenosine monophosphate-activated protein kinase (AMPK). The effect of Nrf2 on downstream proteins is precisely regulated by Kelch-like ECH-related protein 1 (Keap1) [77]. In addition, Rg1 can alleviate H_2_O_2_-induced oxidative stress injury in N2a cells by activating the Nrf2/HO-1 signaling pathway [92]. Furthermore, the ginsenoside CK can activate the Nrf2/Keap1 signaling pathway, which enhances the expression of the downstream HO-1 molecule to deal with oxidative stress and reduce oxidative damage [51]. The decreased superoxide dismutase (SOD) and glutathione peroxidase (GSH-Px) levels and increased malondialdehyde (MDA) and lactate dehydrogenase (LDH) levels caused by Aβ aggregation cause oxidative stress and oxidative damage. CK can increase the levels of SOD and GSH-PX, and reduce the levels of MDA [51]. In addition, Rg1 can induce oxidative stress by increasing the levels of SOD and GSH-PX and decreasing the levels of LDH and MDA [92,93]. Furthermore, Rg3 can increase the levels of SOD, catalase (CAT), and GSH-Px, and decrease the levels of MDA [86]. Rh2 can significantly reverse decreased SOD activity, increased MDA levels, and decreased GSH content induced by scopolamine, contributing to reduced antioxidant stress [79]. PPT can exert antioxidant activity by increasing SOD activity and reducing MDA levels in the hippocampus [81]. GFREH can increase SOD and CAT levels, and decrease MDA and LDH levels in worms, and has an antioxidant role [94].

#### 3.4.2. Anti-Inflammation

**Figure 6:**
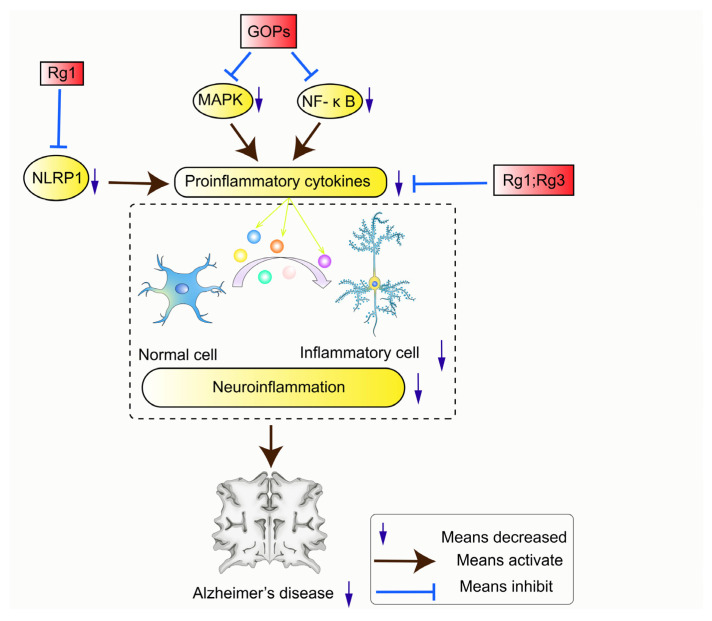
Signaling pathways related to neuroinflammation.

Neuroinflammation is an important cause of neuronal damage and is one of the causative factors in the development of AD. The preclinical, intermediate, and late phases of AD are all accompanied by a persistent process of neuroinflammation [12]. The effect of *P. ginseng* on inflammation is mainly due to the effects of different signaling pathways on inflammatory factors, as shown in Figure 6.

The ginsenoside Rg1 exerts anti-inflammatory effects by inhibiting the activity of the NLRP1 inflammasome and regulating the expression of proinflammatory cytokines (IL-1β and IL-18) [95]. Rg3 can ameliorate the chronic inflammatory state by reducing proinflammatory cytokines, such as TNF-α, IL-1β, COX-2, iNOS, and IL-6 [62,96]. In addition, it is reported that ginseng oligopeptides (GOPs) can reduce the proinflammatory cytokines TNF-α and IL-β, which may be due to the inhibition of genes that cause inflammation (MAPK and NF- κ B) [97].

There are many active substances in *P. ginseng* that exert anti-inflammatory and antioxidant effects through different methods and signaling pathways synergistically, and have been proven to improve cognitive and memory impairment. They can be used as potential drugs for AD.

### 3.5. Prevention of Mitochondrial Damage

**Figure 7:**
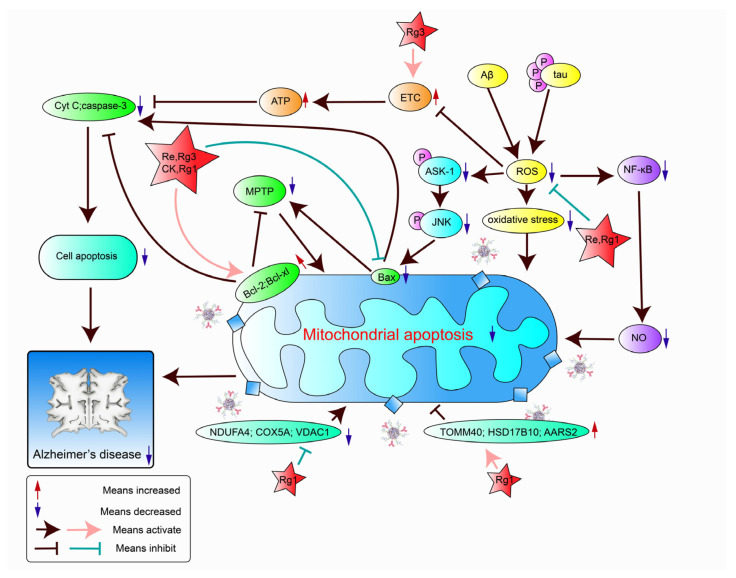
Signaling pathways related to mitochondrial apoptosis.

Studies have shown that mitochondrial dysfunction is a pathological change that usually occurs in the early stages of AD. Mitochondrial damage usually leads to synaptic damage, apoptosis, and neuronal degeneration [98]. The effects of signaling pathways on apoptosis and anti-apoptosis proteins are mainly responsible for *P. ginseng*’s ability to ameliorate mitochondrial damage, as shown in Table 5 and Figure 7.

#### 3.5.1. Relationship of Mitochondrial Damage and Other Pathogenic Mechanisms

There are interactions between mitochondrial damage and other pathogenic factors of AD. Increased Aβ and phosphorylated tau (p-tau) causes increased ROS, as reported by Pradeepkiran et al. [101]. Oxidative stress caused by increased reactive oxygen species or decreased antioxidants can cause mitochondrial DNA damage, loss of integrity, and mitochondrial dysfunction. Mitochondrial dysfunction is often accompanied by abnormal energy metabolism, in which not enough energy is produced for the brain to maintain learning and memory function. In addition, abnormal lipid metabolism causes abnormal energy metabolism, and lipid metabolism can produce more ROS and aggravate mitochondrial damage. Mitochondrial dysfunction can also cause amino acid metabolism disorders and changes to amino acid levels, and affect the occurrence of AD. Thus, the amelioration of mitochondrial damage is a potential target for improving AD.

#### 3.5.2. Prevention of Mitochondrial Damage by Active Compounds of *P. ginseng*

Located on the mitochondrial membrane, Bcl-2 and Bcl-xl are crucial anti-apoptotic substances, while the apoptotic promoter Bax, which physically resembles Bcl-2, can cause cytochrome C (Cyt C) release, caspase-3 expression, and, ultimately, apoptosis [102]. Studies have shown that Bax and Bcl-2 can regulate the permeability of the mitochondrial membrane by modulating the mitochondrial permeability transition pore (MPTP), and can also prevent the release of mitochondrial Cyt C. Bax and Bcl-2 can exert their anti-apoptotic effects by both of the above mechanisms, preventing cell death [86]. For this reason, increasing Bcl-2 and Bcl-xl expression, decreasing Bax and caspase-3 expression, and decreasing Cyt C release are important mechanisms to stop mitochondrial damage. The ginsenoside CK was found to increase Bcl-2 expression and decrease Bax and caspase-3 expression [51]. Disturbances in the electron transport chain (ETC) are an important component of mitochondrial dysfunction. The ETC is an important part of the balance between intracellular ROS and cellular redox states, including NAD+ and NADH. Overproduction of ROS leads to a decrease in ETC activity, which in turn leads to a decrease in ATP synthesis. However, ATP can inhibit Cyt C activity by binding to Cyt C. Therefore, maintaining stable ETC and ATP levels is one way to stop mitochondrial damage. The ginsenoside Rg3 was found to regulate abnormal energy metabolism and restore mitochondrial ETC disorder, thus improving mitochondrial dysfunction in AD rats. In addition, Rg3 can regulate the levels of apoptosis-related factors (Bax and Bcl-2) and prevent mitochondrial damage. Furthermore, the ginsenoside Rg3 was found to improve cognitive and memory impairment induced by mitochondrial dysfunction in AD rats by regulating abnormal purine metabolism, amino acid metabolism, and anti-apoptosis activity [86]. The ginsenoside Re can downregulate Bax expression, increase the Bcl-2/Bax ratio, and suppress Cyt C release by reducing ROS production, decreasing apoptosis signal kinase 1 (ASK-1) and c-Jun N-terminal kinase (JNK) phosphorylation [67]. Upregulation of the NF-κB/NO signaling pathway can also cause mitochondrial damage. The ginsenoside Rg1 can increase the Bcl-2/Bax ratio, reduce the release of Cyt C from mitochondria to the cytoplasm, and block mitochondria-mediated apoptosis by scavenging ROS to downregulate the NF-κB/NO signaling pathway and inhibit NO production [99]. In addition, Rg1 can change a variety of mitochondrial proteins, which can reduce AD-related mitochondrial damage and produce a certain therapeutic effect. Mitochondrial proteins containing hydroxysteroid 17-beta dehydrogenase 10 (HSD17B10), alanyl-tRNA synthetase 2 (AARS2), translocase of outer mitochondrial membrane 40 (TOMM40), Cyt C oxidase subunit 5A (COX5A), voltage-dependent anion channel 1 (VDAC1), and renamed COXFA4 (NDUFA4) are associated with AD-related mitochondrial dysfunction. It has been reported that Rg1 prevents mitochondrial damage by upregulating mitochondrial proteins HSD17B10, AARS2, and TOMM40, and downregulating NDUFA4, VDAC1, and COX5A [100].

### 3.6. Regulation of Gut Microbiota

In recent years, studies have found a causal relationship between the gut microbiota and AD [103]. Imbalances in the gut microbiota may promote Aβ aggregation, affect neurotransmitter levels, and trigger neuroinflammation; this may be an indirect factor in the development of AD. Active substances in *P. ginseng*, such as ginsenosides and polysaccharides, have been found to regulate intestinal microbiota and neurotransmitter disorders caused by them; however, the mechanism by which *P. ginseng* regulates gut microbe disorders to improve neuroinflammation and Aβ aggregation has not yet been elucidated, as shown in Table 6.

#### 3.6.1. Regulation of Gut Microbiota

There are some healthy microorganisms in the gut that can reduce neuroinflammation, vascular pathology, and Aβ aggregation, and the maintenance of brain balance plays a beneficial role in the treatment of AD [108]. However, under certain pathological conditions, the intestinal microenvironment may be more conducive to the overgrowth of some bacteria. GRg1 may alter the composition and abundance of gut microbes to improve AD [105]. Dushen tang (a ginseng decoction), which is composed of a single herbal material, *Panax ginseng* C.A. Meyer, could improve disturbances of the gut microbiota, including *Bacteroidales*, *Lactobacillus*, and *Bacteroides*, to improve memory impairment [107].

#### 3.6.2. Gut Microbes and Aβ Aggregation

Gut microbe imbalances can promote lipopolysaccharide and amyloid secretion, and accelerate Aβ aggregation and inflammation [109]. A study found that the gut microbiota may stimulate the MAPK signaling pathway in the brain and promote amyloid deposition [110]. The secretion of lipopolysaccharides and amyloid proteins can interfere with the permeability of the intestinal tract and the blood–brain barrier [111]. Increased permeability of the gut and the blood–brain barrier can also allow pathogens to reach the nervous system, and may induce AD [112].

#### 3.6.3. Gut Microbes and Regulation of Neurotransmitters

In addition, increased intestinal and blood–brain permeability affects the production and absorption of neurotransmitters, such as serotonin, GABA, kynurenine, and catecholamine [113]. Intestinal microorganisms produce neuroactive substances that affect neuronal function, host metabolism, and immunity, and then affect the neural pathways of the intestinal tract and nervous system, which has an impact on the occurrence of AD [114]. In addition, it has been found that disorders of the gut microbiota in AD patients affect tryptophan metabolism, with an increased kynurenine-to-tryptophan ratio (Kyn/Trp) [115]. *P. ginseng* polysaccharides were found to increase the microbial metabolite valeric acid and reduce the proportion of kynurenic acid and Kyn/Trp. However, further research is needed to determine whether they can have therapeutic effects on AD through this pathway [106]. The ginsenoside Rb1 can significantly increase the relative abundance of specific probiotics, especially *Lactobacillus helveticus*. Increased levels of *Lactobacillus helveticus* were found to upregulate the expression of GABAA (α2, β2, and γ2) and GABAB (1b and 2) receptor subunits in the hippocampus and striatum of rats, thereby exerting a neuroprotective effect [104].

#### 3.6.4. Gut Microbes and Neuroinflammation

Bidirectional communication between the gut microbiota and neuroactive substances via the gut–brain axis may be involved in inflammation. Some components of the intestinal microbiota can synthesize and release cytokines to activate inflammatory signals [116]. The intestinal microbiota may also regulate the kynurenine pathway (KP) and then neuroinflammation [117]. The metabolites produced by some intestinal microorganisms can affect the activity of the microglia, further mediate neuroinflammation, and cause neuronal necrosis, and can potentially play a role in the occurrence of AD [118].

In recent years, gut microbes have occupied an important place in disease research. Studies have also identified an important role for gut microbes in the development of AD. *P. ginseng* and its active ingredients have been found to regulate intestinal flora disorders, but the specific mechanism of action still needs more in-depth research.

## 4. Clinical Studies on *P. ginseng* to Improve AD

To elucidate the feasibility of applying *P. ginseng* clinically, we examined some clinical evidence justifying the importance of studying *P. ginseng* for the treatment of AD.

### 4.1. Signs and Symptoms of AD

Patients with AD often present with cognitive deficits, loss of independence, and difficulty maintaining a normal life. Clinically, they present with Aβ protein accumulation in the cerebrospinal fluid and hyperphosphorylation of tau. In addition, temporal lobe and hippocampal atrophy may be a marker for the development of AD [119].

### 4.2. Current Diagnosis of AD

Initially, the onset of AD was diagnosed primarily by the presence of dementia, with the AD Assessment Scale (ADAS) and mini-mental state examination (MMSE) used for diagnosis [120]. However, a minority of AD patients do not have prominent memory impairment. Therefore, with the continuous development of related research, the diagnosis of AD has changed from the presence of dementia symptoms to the presence of biomarkers. Biomarkers have been categorized as A (Aβ), T (P-tau), and N (neurodegeneration), in what is known as the ATN framework [119].

### 4.3. Clinical Studies on P.ginseng to Improve AD

In addition to animal and cell experiments, based on the ADAS and MMSE, some clinical evidence has shown that *P. ginseng* can improve cognitive impairment, including a report by Lee [121] et al. showing that *P. ginseng* was able to improve cognitive performance in AD patients. In addition, a *P. ginseng* product made by high-temperature heat treatment, sun ginseng-135, was found to improve cognitive deficits in patients with clinical AD [120]. Despite the evidence that *P. ginseng* improves cognitive deficits in AD patients, the mechanism of action on ATN biomarkers and other mechanisms have not been investigated. Therefore, more in-depth research is needed in order to truly apply *P. ginseng* to clinical use.

## 5. Application of Spatial Metabolomics in AD Research

Spatial metabolomics technology is widely used in various fields and has received extensive attention in the field of medicine. It can be used to detect differences in the distribution of active ingredients in the tissues of herbs. It has played an important role in the treatment of nervous system diseases, including AD, and can be used to detect spatial and quantitative changes in neurotransmitters and Aβ levels. The study of the protein deposition process and early changes in lipid metabolism can also elucidate the distribution of therapeutic drugs in order to understand their mechanism for treating diseases. The discovery of spatial metabolomics is of great significance in the study of AD, and has fostered significant progress in the field.

### 5.1. Spatial Metabolomics and P. ginseng

Mass spectrometry and imaging technology can be used to analyze the chemical components of different parts of herbs and are important in the study of the distribution of active substances in different parts of *P. ginseng*. Ultra-high-performance liquid chromatography quadrupole/time-of-flight mass spectrometry (UPLC-QTOF MS) and DESI-MSI have been used to analyze *P. ginseng*, and PPT, PPD, and other compounds in *P. ginseng* have been used as biomarkers for identifying the years of *P. ginseng*. In addition, the distribution of some malonyl ginsenosides, the neutral ginsenoside Rg1, and other components in different tissues of *P. ginseng* has been described [122].

### 5.2. Spatial Metabolomics and the Diagnosis of AD

Spatial metabolomics is widely used in the field of medicine. It can be used for the detection of pathological markers [123] and the study of disease pathogenesis and treatment mechanisms. Spatial metabolomics technology plays an important role in the study of the pathogenesis and treatment mechanisms of a variety of diseases, including cancer [124], cardiovascular diseases [125], tumors [126], diabetes nephropathy [127], and nervous system diseases. Spatial metabolomics plays an important role in the study of depression [127], AD, Parkinson’s disease [128], diabetes encephalopathy [129], and other neurological diseases. It can be used to detect changes in the content and spatial distribution of Aβ, neurotransmitters, and lipids during the initiation of AD treatment. It can also be used to visualize the mechanisms and sites of action of AD treatment drugs. AFADESI-MSI and metabolomics analyses allow the mapping of hundreds of metabolites with different polarity functions involving different metabolic pathways. These metabolites include neurotransmitters, organic acids, purines, and carbohydrates. Thus, AFADESI-MSI and metabolomics could be applied to the discovery of dysfunctional metabolites in brain microregions based on the pathological study of a scopolamine treatment model of AD, which could provide spatial information on the metabolic events of diseases [130]. Amyloid plaques are an early manifestation of AD. Mass spectrometry imaging technology can be used to visualize the formation of plaques and peptide deposition in different structures of the brain and to understand the characteristics of early plaque formation, which can promote research on the early prevention of AD [131]. Mass spectrometry imaging can also be used to visualize the distribution and metabolism of neurochemicals in the brain caused by drugs. DESI-MSI was used to study the effects of *P. ginseng* and American ginseng on the distribution of brain neurochemicals in rats, with neurochemical substances related to the cold and warm characteristics of the two plants screened out. They were divided into warm markers, which promote energy metabolism in the body, improve the function of the endocrine system, and enhance central nervous system excitability, as well as cool markers, which reduce central nervous system excitability, weakening the metabolism and stress response. These can assist in the treatment and research of nervous system diseases [132]. Lipid metabolism dysfunction is closely related to AD pathogenesis. MALDI-MSI can be used to intuitively reveal the spatial distribution and metabolism of mouse brain regions. It can help in understanding lipid changes in the early stages of AD [133].

### 5.3. Spatial Metabolomics and Drug Applications

Spatial metabolomics can also help to understand the metabolic distribution of drugs in the body [134].

Spatial metabolomics plays an important role in the study of drug metabolism in vivo. LC-MS/MS combined with nano-spray DESI mass spectrometry was used to analyze the drug distribution of the ginsenoside Rg1 in rats administered with intravenous Rg1 at different times, and the distribution and content changes in the kidney, liver, lung, spleen, heart, and brain at different times were obtained, providing a theoretical basis for drug development [135].

In summary, spatial metabolomics is expected to serve as technical support when studying the mechanisms of action and the pharmacological basis of various active ingredients in *P. ginseng* in the treatment of AD.

## 6. Conclusions

*P. ginseng* contains various active ingredients, including ginsenosides, polysaccharides, and gintonin. These bioactive compounds have multiple pharmacological activities and are effective in improving AD.

The pathogenesis of AD includes Aβ accumulation, tau hyperphosphorylation, increased neurotransmitter levels, oxidative stress, neuroinflammation, mitochondrial apoptosis, and disordered gut microflora. The effects of each pathogenetic factor in AD are related to different signaling pathways. The components in *P. ginseng* contribute to the treatment of AD by acting on different targets and pathways. In this article, we constructed a signaling pathway network related to the action of active ingredients in *P. ginseng* and the occurrence of AD to explain the relevant processes involved in using *P. ginseng* for AD treatment and to provide a theoretical basis for developing it as a therapeutic herb for this disease. However, some of these mechanisms have not been fully elucidated and further studies are still needed, especially regarding the specific mechanisms by which *P. ginseng* improves AD by modulating gut microbe disorders. Therefore, based on the current evidence, the pathogenesis of AD and the mechanism of action of *P. ginseng* should be studied more thoroughly in the future to promote *P. ginseng* and maximize its anti-AD effects.

Evidence from clinical studies has also shown that *P. ginseng* can improve AD; however, the specific mechanism of action has not been elucidated. Therefore, adequate research is still needed to realize the real application of *P. ginseng*.

Spatial metabolomics can be applied to the study of the early pathological components of AD, including neurotransmitters and Aβ. In addition, it can be used detect the distribution of effective ingredients in *P. ginseng* after administration and its regulatory effect on neuroactive substances. Therefore, it is believed that spatial metabolomics can provide technical support for the more effective development of *P. ginseng* as a medicinal herb for the treatment of AD.

## Figures and Tables

**Table 1:** The effects of *P. ginseng* on the pathogenesis of AD related to Aβ.

*P. ginseng* Compounds	Experimental Model	Effects	Ref.
Ginsenoside CK	ICR mice	Suppress BACE1 and PS1; enhance IDE	[51]
Ginsenoside Rg1	Sprague-Dawley rats	Suppress APP, CDK5, P-PPARγ, and BACE1; enhance IDE	[52]
Ginsenoside Rb1	PC12 pheochromocytoma cells	Enhance PPARγ	[53]
Ginsenoside CK, F1, Rh1 and Rh2	Molecular docking	Suppress BACE1	[54]
Ginsenoside F1	Mouse neuroblastoma neuro-2a (N2a) cells and human neuroblastoma SH-SY5Y cells	Enhance IDE and NEP	[55]
Ginsenoside Rg3	SK-N-SH cells	Enhance NEP	[56]
Ginsenoside Rg1	Wild type (WT) and APP/PS1 AD mice	Suppress NOX2, ROS, APP, and BACE	[57]
Ginsenoside Rd	*C. elegans*	Suppress ROS and MAPK	[58]
Ginsenoside Re	N2a/APP695 cells	Enhance PPARγ; suppress BACE1	[59]
Ginsenoside Rb1, Rb2	Molecular docking	Suppress BACE1	[60]
DDPU	SH-SY5Y cells, APP/PS1 transgenic mice, CHO-APP cells, HEK293-APP swe cells, primary neurons, or primary astrocytes	Suppress BACE1, PERK, P-eIF2 α, PI3K, P-AKT, P-mTOR, and P-ULK1	[61]
Ginsenoside Rg3	Neuro-2a (N2a) murine neuroblastoma and HMO6 human microglial cells	Enhance SRA	[62]
A 4.7-kDa ginseng-derived polysaccharide (GP4)	SH-SY5Y cells and *C. elegans*	Enhance PINK1 and Parkin	[63]
combined ginsenosides (SumI)	AD worms	Enhance HSPs and HSF-1	[26]
Ginsenoside F1	APPswe/PSEN1dE9 double-transgenic AD mice with a B6 × C3 background and B6 × C3 wild type mice	Enhance P-CREB and BDNF	[64]

**Table 2:** The effects of *P. ginseng* on the pathogenesis of AD related to tau proteins.

*P. ginseng* Compounds	Experimental Model	Effects	Ref.
Ginsenoside Rb1	ICR mice	Suppress pGSK-3; enhance PP2A	[72]
Ginsenoside Rd	SD rats	Enhance PP2A	[73]
Ginsenoside Rg1	Wistar rats	Enhance PP2A	[74]
Ginsenoside Rb1	SD rats	Suppress calpain, CDK5, and P25; enhance P35	[75]

**Table 3:** The effects of *P. ginseng* on the pathogenesis of AD related to neurotransmitters.

*P. ginseng* Compounds	Experimental Model	Effects	Ref.
Ginsenoside Rh2	Male ICR mice	Enhance ACh	[79]
Ginsenoside Rg1	Adult male Sprague-Dawley rats	Enhance ACh	[68]
GSLS	Cell	Suppress AChE	[80]
PPT	Male Institute of Cancer Research mice	Enhance ACh	[81]
Ginsenoside Re, Rg3	In-vitro enzyme assays	Suppress AChE and BChE	[60]
Ginsenoside Rb1	Male Sprague-Dawley rats	Enhance ACh	[82]
Ginsenoside Re	Adults male Sprague-Dawley rats	Enhance DA and ACh	[83]
Ginsenoside Rg3	Male C57BL/6 mice	Suppress AChE	[84]
Gintonin	Male ICR or C57BL/6 mice	Enhance ACh and ChAT; suppress AChE	[85]
Ginsenosides	Male Wistar rats	Enhance GABA, ACh, DA, Gly, and 5-HT; suppress Glu and Asp	[76]
Ginsenoside Rg3	Male Wistar rats	Improve Ala, Asp, Glu, D-Gln, D-Glu, and Trpab normal metabolism	[86]

**Table 4:** The effects of *P. ginseng* on the pathogenesis of AD related to oxidative stress and neuroinflammation.

*P. ginseng* Compounds	Experimental Model	Effects	Ref.
Ginsenoside CK	ICR mice	Enhance Nrf2, keap1, HO-1, SOD, and GSH-Px; suppress MDA	[51]
Ginsenoside Rk3	APP/PS1 mice, PC12 cells	Suppress ROS; enhance P-AMPK, Nrf2, HO-1, NQO1, and Keap1	[77]
Ginsenoside Rg1	Mouse neuroblastoma N2a cells	Enhance Nrf2, HO-1, and SOD; suppress MDA and LDH	[92]
Ginsenoside Rg1	Male APP/PS1 mice	Suppress ROS and NOX2	[57]
Ginsenoside Rg1	C57BL/6 mice, mice NSCs	Enhance SOD and GSH-Px; suppress MDA, ROS, Akt, and mTOR	[93]
Ginsenoside Rg3	Male Wistar rats	Enhance SOD, CAT, and GSH-Px; suppress MDA and ROS	[86]
Ginsenoside Rh2	Male ICR mice	Enhance SOD and GSH; suppress MDA	[79]
PPT	Male Institute of Cancer Research mice	Enhance SOD; suppress MDA	[81]
GFREH	*C. elegans*	Enhance SOD and CAT; suppress MDA, LDH and ROS,	[94]
Ginsenoside Rg1	Male ICR mice	Suppress NLRP1 and proinflammatory cytokines (IL-1β and IL-18)	[95]
Ginsenoside Rg3	Male Sprague-Dawley (SD) rats	Suppress proinflammatory cytokines (TNF-α, IL-1β, and COX-2)	[96]
Ginsenoside Rg3	Neuro-2a (N2a) murine neuroblastoma and HMO6 human microglial cells	Suppress proinflammatory cytokines (iNOS, IL-6, and TNF-α)	[62]
Ginseng oligopeptides (GOPs)	Male Sprague-Dawley (SD) rats	Suppress MAPK, NF-κ B, and proinflammatory cytokines (TNF-α IL-β)	[97]

**Table 5:** The effects of *P. ginseng* on the pathogenesis of AD related to mitochondrial apoptosis.

*P. ginseng* Compounds	Experimental Model	Effects	Ref.
Ginsenoside CK	ICR mice	Enhance Bcl-2; suppress Bax and caspase-3	[51]
Ginsenoside Rg3	Male Wistar rats	Improve ETC and ATP; enhance Bcl-2 Suppress Cyt C, Bax, and ROS	[86]
Ginsenoside Re	SH-SY5Y human neuroblastoma cells	Suppress Bax, ROS, ASK-1, and JNK	[67]
Ginsenoside Rg1	Primary cultured cortical neurons were prepared from embryonic day (D17-18) Sprague-Dawley (SD) rat fetuses	Enhance Bcl-2; suppress NF-κB, NO, ROS, and Bax	[99]
Ginsenoside Rg1	SH-SY5Y cells	Enhance HSD17B10, AARS2, and TOMM40; suppress NDUFA4, VDAC1, and COX5A	[100]

**Table 6:** The effects of *P. ginseng* on the pathogenesis of AD related to gut microbiota.

*P. ginseng* Compounds and Classic Chinese Formulation	Experimental Model	Effects	Ref.
Ginsenoside Rb1	Sprague-Dawley rats	Enhance GABAA (α2, β2, and γ2), GABAB (1b and 2), *Bifidobacterium longum*, *Bifidobacterium dentium*, *Lactobacillus brevis*, *Lactobacillus helveticus*, and *Lactobacillus rhamnosus*	[104]
Ginsenoside Rg1	Male conventional tree shrews	Alter the composition and abundance of gut microbiota	[105]
Ginseng polysaccharides (GPs)	C57BL/6J mice	Increase valeric acid and reduce L-canine uric acid and Kyn/Trp	[106]
Dushen Tang	Adult male Sprague-Dawley rats	Correct the disturbance of the gut microbiota	[107]

## Data Availability

Data are contained within the article.
